# Pembrolizumab-induced Hemophagocytic Lymphohistiocytosis: an immunotherapeutic challenge

**DOI:** 10.1186/s41199-020-0050-3

**Published:** 2020-02-03

**Authors:** James Kalmuk, Jon Puchalla, Gong Feng, Anshu Giri, John Kaczmar

**Affiliations:** 1 Department of Internal Medicine, Medical University of South Carolina, 96 Jonathan Lucas Street, Charleston, SC 29425 USA; 2 Department of Pathology and Laboratory Medicine, Medical University of South Carolina, 171 Ashley Avenue, Charleston, SC 29425 USA; 3 Department of Hematology/Oncology, Medical University of South Carolina Walton Research Building, 39 Sabin Street, Charleston, SC 29425 USA

**Keywords:** Hemophagocytic Lymphohistiocytosis, HLH, Pembrolizumab, Head and neck squamous cell carcinoma, Immune-related adverse events, irAEs, Checkpoint inhibitors

## Abstract

**Background:**

As the number of indicated malignancies for which immune checkpoint inhibitor therapy such as pembrolizumab grows the descriptions of associated immune-related adverse events (irAEs) increases as well. On rare occasions immunotherapy can lead to development of Hemophagocytic Lymphohistiocytosis (HLH) which is a potentially lethal inflammatory disorder characterized by histiocyte activation and cytokine storm. At this time no cases of HLH developing in head and neck squamous cell carcinoma (HNSCC) patients receiving pembrolizumab have been reported.

**Case presentation:**

Here we describe the first documented case of pembrolizumab-induced HLH in a 61 year-old male with metastatic HNSCC after having received multiple prior cycles of pembrolizumab without event. Following cycle 14 the patient developed fever associated with new pancytopenia and transaminitis prompting hospital admission. Infectious workup was negative, his metastatic lesions were found to be stable, and there was no evidence of new malignancy. Further workup demonstrated hyperferritinemia and bone marrow biopsy demonstrated hemophagocytosis concerning for pembrolizumab-induced HLH. Etoposide and dexamethasone therapy was initiated leading to clinical improvement and safe discharge.

**Conclusions:**

Immunotherapy is a groundbreaking therapeutic intervention for patients with malignancy, however by nature of their mechanism carry a risk of inflammatory side effects. In rare circumstances these inflammatory reactions include potentially deadly syndromes such as HLH. As immunotherapeutics such as pembrolizumab become more widely utilized increased awareness of complications such as HLH is clinically relevant.

## Background

Checkpoint inhibitors such as pembrolizumab are an innovative therapeutic intervention for patients with an ever-expanding number of malignancies and have led to profound clinical responses. However, this same class of drugs carries a significant risk for inflammatory side effects known as immune-related adverse events (irAEs). As an unintended consequence of their mechanism wherein they are thought to enhance cytotoxic T-cell activity against cancer cells they may also promote the pathologic hyperinflammatory state known as Hemophagocytic Lymphohistiocytosis (HLH). Here we present the first documented case of HLH arising secondary to pembrolizumab therapy in a patient with metastatic head and neck squamous cell carcinoma (HNSCC) who responded well to classical HLH therapy including dexamethasone and etoposide. By presenting this case we aim to increase awareness of potentially lethal complications of immunotherapy given the increasing number of indications for checkpoint inhibitors such as pembrolizumab.

## Case presentation

A 61 year-old gentleman with a history of p16+ squamous cell carcinoma of the oropharynx with metastatic spread to bilateral lungs initially received cisplatin and radiotherapy with subsequent transition to clinical trial involving 6 weeks of anti-platelet therapy (aspirin/clopidogrel) with concurrent pembrolizumab followed by pembrolizumab monotherapy after completion of the trial. He demonstrated stable disease while on treatment with excellent tolerance to immunotherapy over his first nine months of treatment with no significant irAEs.

Four days following cycle 14 of pembrolizumab the patient developed fever > 39.5 °C and malaise prompting a visit to his primary care provider who prescribed a course of oral antibiotics without symptomatic improvement. He subsequently presented to his Oncology clinic, was found to have a new pancytopenia and transaminitis as well as tender hepatomegaly on exam concerning for autoimmune hepatitis, and was admitted to the hospital for methylprednisolone therapy. Admission bloodwork compared to most recent values from 3 weeks prior is presented in Table 1.

**Table 1:** Admission Blood Counts and Liver Function Testing

	Admission	3 weeks prior to admission	Normal range
White Blood Cell (WBC)	3.37	6.69	4.8–10.8 K/cumm
Hemoglobin	9.5	12.6	14–18 g/dL
Platelets	32	195	140–440 K/cumm
Aspartate Aminotransferase (AST)	289	20	5–34 U/L
Alanine Aminotransferase (ALT)	474	20	5–45 U/L
Alkaline Phosphatase (ALP)	474	76	35–150 U/L
Total Bilirubin	1.8	0.5	0.2–1.2 mg/dL

**Fig. 1:**
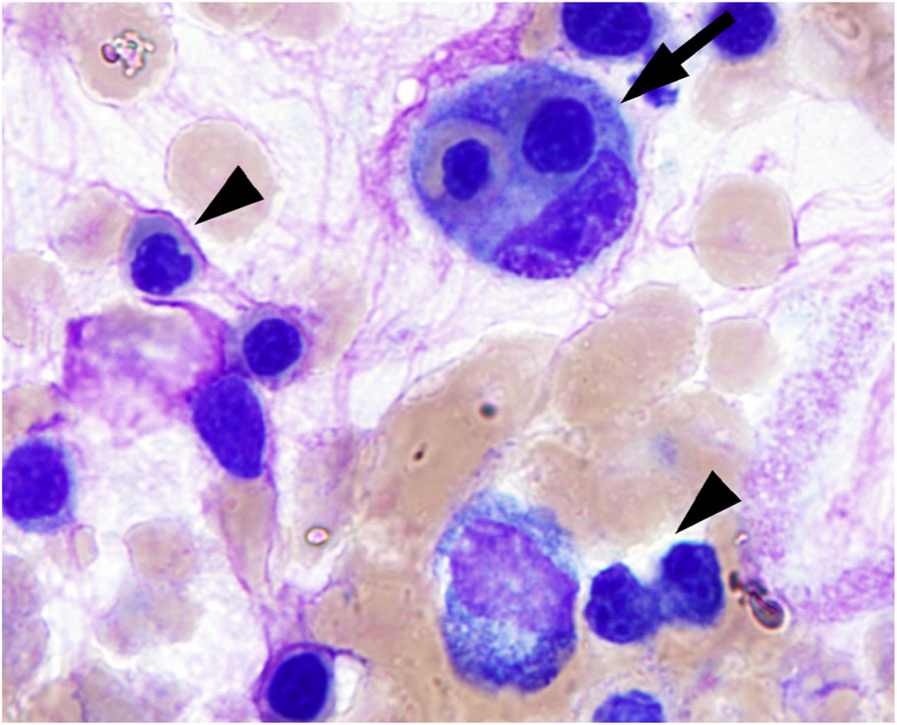
Bone marrow aspirate shows hemophagocytic histiocytes (black arrow) and dyserythropoiesis (irregular nuclear contour and internuclear-bridging, black arrow-head)

Infectious workup including acute hepatitis panel was negative while haptoglobin was within normal limits. Peripheral smear demonstrated pancytopenia with rare schistocytes. Abdominal CT demonstrated periportal edema and gallbladder wall thickening consistent with acute hepatitis as well as new splenomegaly. Due to progressive pancytopenia despite methylprednisolone bone marrow biopsy was performed demonstrating widespread hemophagocytosis (Fig. 1) while additional workup revealed ferritin 57,934 ng/mL (normal range 22–322 ng/mL), fibrinogen 134 mg/dL (normal range 231–486 mg/dL), triglycerides 285 mg/dL (normal range < 150 mg/dL), soluble interleukin-2 receptor (sIL-2R) 79,600 pg/mL (normal range < 1033 pg/mL), and an absolute Natural Killer (NK) cell population of 2 cells/μL (normal range 75–599 cells/μL) concerning for pembrolizumab-induced HLH. The patient was subsequently transitioned to dexamethasone 10 mg/m^2^ and etoposide 150 mg/m^2^ per published guidelines for HLH [1].

**Fig. 2:**
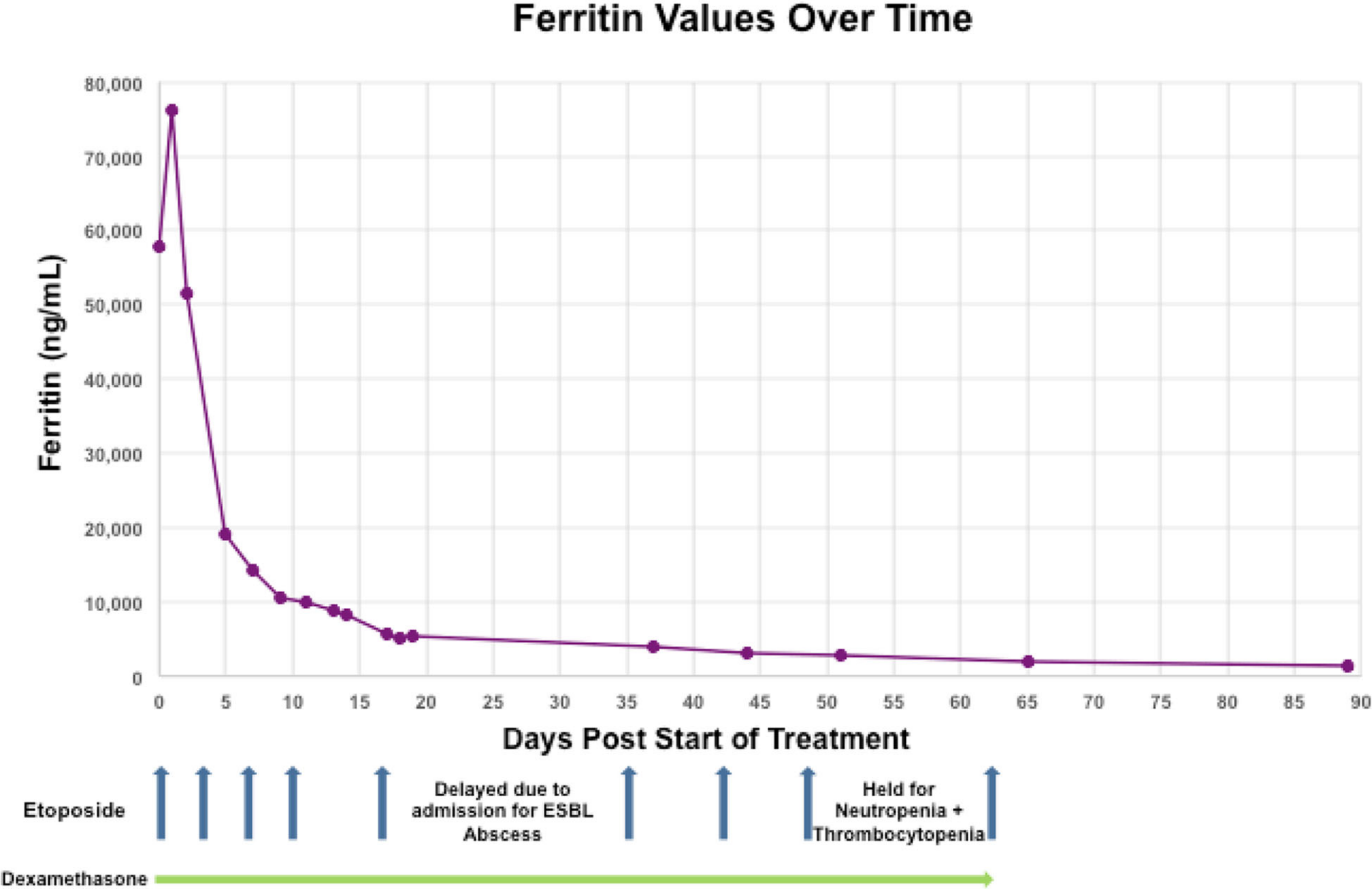
Ferritin Values Over Time

The patient’s hospital course was complicated by neutropenia with eventual count improvement allowing for safe discharge on dexamethasone taper and outpatient etoposide infusions. Ferritin was trended as a marker of treatment response as demonstrated in Fig. 2.

The patient’s outpatient course was notable for an ESBL soft-tissue abscess requiring hospital admission for incision/drainage and antibiotics, which delayed his 4th week of etoposide. In addition, his 7th week dose of etoposide was held due to transient neutropenia and thrombocytopenia. He otherwise completed a standard HLH regimen and by the time of restaging scans 3 months after HLH diagnosis he had returned to his woodworking business, regained all lost weight (~ 11.5 kg), and demonstrated normalization of his liver function testing as well as improvement in his WBC and platelet counts. Initial restaging scans demonstrated stable pulmonary metastatic disease and no new visceral metastases. The patient was monitored off immunotherapy for 8 months without disease progression before developing progressive mediastinal lymphadenopathy and a painful ischial metastatic lesion. He otherwise exhibited unchanged pulmonary disease. The patient underwent radiation to the osseous lesion with clinical improvement and pembrolizumab was restarted. To date the patient has received 5 cycles pembrolizumab since immunotherapy re-initiation with interval reduction in lymph node size, stable pulmonary metastases, no new or progressive bony disease, and no clinical evidence of recurrent HLH.

## Discussion

HLH is an uncommon yet life-threatening hematologic disorder characterized by aberrant immune-cell activation, pro-inflammatory cytokine release, and multi-organ dysfunction. HLH is diagnosed based on meeting at least 5 of 8 criteria set forth by the Histiocyte Society representing common pathologic findings in patients with HLH [1]. These criteria include fever > 38.5 °C, splenomegaly, 2 or more cytopenias (hemoglobin < 9 g/dL, platelets < 100 × 10 g/L, neutrophils < 1 × 10 g/L), hypertriglyceridemia > 265 mg/dL and/or hypofibrinogenemia < 150 mg/dL, hemophagocytosis, decreased NK cell activity, hyperferritinemia > 500 ng/mL, and sIL-2R > 2400 U/mL [1]. Other manifestations include encephalopathy, respiratory failure, coagulopathy, and hepatitis, which was an initial concern with our patient [2]. HLH is subdivided into both primary and secondary types. Primary HLH typically arises in the pediatric population due to genetic mutations causing dysfunction in cytotoxic granule trafficking and perforin activity which in turn leads to immune hyperactivation and systemic inflammation [3]. Secondary HLH is described in adults with no known underlying mutational predisposition and is triggered by infection, malignancy, or rheumatologic disease (where it is referred to as Macrophage Activation Syndrome (MAS)). More recently, there has been proposal of an additional form of HLH known as immune-related HLH caused by immunotherapy leading to aberrant T-cell activation, particularly CD8+ T-cells [3, 4].

Immunotherapy represents a revolutionary cancer treatment modality that via a wide array of mechanisms increases immune cell activity against tumor cells. Two programmed cell death-1 (PD-1) receptor inhibitors, nivolumab and pembrolizumab, are approved for the treatment of recurrent/metastatic HNSCC. PD-1 is expressed by immune cells and helps regulate self-tolerance by downregulating the immune response. Binding of PD-1 by PD-1 ligand (PD-L1) in the tumor microenvironment compromises normal T-cell function and may promote conversion of cytotoxic T-cells into T-regulatory cells [3]. Interfering with PD-1 and PD-L1 signaling with these checkpoint inhibitors may increase T-cell cytotoxic activity and engender tumor regression, however due to the broad nature of these interactions there are implications for both cancer cells as well as the normal tissues of the host. A wide array of potential inflammatory side effects from immunotherapy-induced activation of T-cells against patient self-antigens have been described and in the literature are known as irAEs [5]. These adverse events range in severity from mild and self-limiting to life-threatening with the majority of described events affecting the pulmonary, gastrointestinal, endocrine and dermatologic systems [5]. As a result, routine bloodwork for patients on immunotherapy often includes complete blood counts, liver function testing, and thyroid function testing both while on treatment as well as for the initial months following treatment completion [3].

Hematologic side effects of immunotherapy are less commonly described however reviews of immunotherapeutic complications report the most common hematologic aberrations are hemolytic anemia and immune thrombocytopenic purpura [5]. On very rare occasions patients receiving immunotherapy may develop HLH. For patients who develop HLH the most commonly implicated agents were those that target cytotoxic T-lymphocyte associated antigen-4 (CTLA-4) as opposed to PD-1/PD-L1 [5]. When compared to other hematologic irAEs HLH occurred earlier and was characterized by increased mortality [5]. HLH has also been described in patients treated with chimeric antigen receptor T-cell (CAR-T) therapy, further highlighting the association between modulation of T-cell activity and development of HLH [6]. Whereas the infectious, malignant, and autoimmune causes of secondary-HLH are due to an underlying disease process, immunotherapy-induced HLH creates a different type of clinical challenge; the patient was started on immunotherapy in an effort to increase T-cell activity against the tumor however an unwanted side effect of this intervention was creation of the pathologic hyperinflammatory state that characterizes HLH. Murine and human models of tumor cells have offered a potential explanation for the association between checkpoint inhibitors and HLH as researchers have observed an inverse correlation between PD-1 expression and tumor-associated macrophage phagocytic activity as well as increased cancer cell phagocytosis by macrophages following PD-1/PD-L1 targeting [3].

On literature review, the case reports discussing immunotherapy-induced HLH have described a wide range of underlying malignancies including Squamous Cell Carcinoma of the Lung, Urothelial Carcinoma, Thymic Carcinoma, Merkel Cell Carcinoma, Breast Cancer, and Melanoma however to our knowledge this is the first report of immunotherapy-induced HLH in a HNSCC patient [3–11]. The time to HLH onset after starting immunotherapy has been shown to be highly variable, ranging from less than a month after receiving either pembrolizumab, nivolumab, or combination ipilimumab/nivolumab to over a year in a patient treated with pembrolizumab [3, 4, 7]. Interestingly, some case reports discuss patients who became critically ill requiring ICU management due to complications of HLH who not only recovered with steroids but also demonstrated tumor regression, with some suggesting that the degree of antitumor efficacy with checkpoint inhibitors may correlate with the severity of side effects patient’s experience [4, 9]. Specific risk factors for development of HLH related to immunotherapy are currently unknown, however one case report describes a patient with breast cancer who developed HLH from pembrolizumab, recovered following a course of steroids, and underwent molecular genetic testing with subsequent discovery of a PRFA91V polymorphism [6]. Such polymorphisms are implicated in primary HLH and are known to exist in up to 3% of the North American population suggesting a potential component of genetic predisposition to develop HLH in adults receiving immunotherapy [6].

The optimal management of irAEs continues to evolve with organizations like the Society for Immunotherapy of Cancer (SITC), National Comprehensive Cancer Network (NCCN), and American Society for Clinical Oncology (ASCO) recently publishing management guidelines [12, 13]. Generally, discontinuing the presumed inciting agent and initiation of corticosteroids are first line treatments [6, 12, 13]. For more severe hyperinflammatory manifestations of immune therapy such as HLH corticosteroid monotherapy may be insufficient. Unfortunately, not only is there little published data to assist with treating side effects of checkpoint inhibitors, there is also a lack of data to guide treatment of secondary HLH in adults with most recommendations coming in the form of expert opinion and the HLH-94/HLH-2004 protocols devised for pediatric patients with primary HLH [1, 6, 14]. These protocols outline a multi-drug regimen including dexamethasone, etoposide, cyclosporine A, and IVIG [14]. Dexamethasone is the preferred corticosteroid for HLH given increased CNS penetration compared to prednisone with an additionally prolonged half-life [15]. Etoposide is a topoisomerase inhibitor that is believed to induce selective apoptosis of pathologically activated T-cells with concurrent reduction in pro-inflammatory cytokine levels [15]. Interestingly, of the few case reports of immunotherapy-induced HLH some patients improved with steroids alone and did not require etoposide [3, 6].

The treatment armamentarium for irAEs such as HLH is rapidly expanding with increasing observational evidence and numbers of clinical trials evaluating cytokine and immune modulation with targeted agents. Daclizumab (anti-CD25 monoclonal antibody) has been described as having successfully treated secondary HLH with the proposed mechanism being depletion of pathologically-activated T-cells that demonstrate increased CD25 expression [16, 17]. Other authors describe successful treatment of refractory HLH by targeting T-cells with anti-CD52 agents such as alemtuzumab [18]. Targeting inflammatory pathways with anakinra (anti-IL-1R) has also been documented as demonstrating therapeutic efficacy in patients with MAS refractory to steroids and cyclosporine A, while in preclinical murine models ruxolitinib (JAK1/2 inhibitor) blocks downstream pro-inflammatory IL and interferon (IFN) signaling leading to amelioration of clinical signs and symptoms of HLH [19, 20]. Others have reported successful treatment of HLH through targeting of the pro-inflammatory cytokine, IL-6, with an antibody directed against it, tocilizumab [21]. The addition of tocilizumab to classical HLH therapy (NCT02007239) is under investigation. Successful HLH therapy is reflected in reduction of inflammatory markers including plasma ferritin levels following treatment initiation, which was seen both in the current case as well as other reports of HLH highlighting the potential usefulness of ferritin as a biomarker for treatment response [3, 9].

In addition to studies evaluating novel therapeutics for irAEs there is increasing interest in understanding the safety and efficacy of rechallenging patients with immunotherapy following irAE resolution. To this end, a recent cohort study of patients with lung cancer who developed irAEs evaluated restarting immunotherapy once the initial event had resolved; retreated patients who did not have a tumor response prior to the irAE demonstrated increased overall survival compared to those that were not retreated while retreated patients who exhibited tumor response prior to the irAE demonstrated similar overall survival to those that were not retreated [22]. As previously noted, our patient was restarted on pembrolizumab due to development of progressive lymphadenopathy and new osseous disease and after 5 cycles thus far has demonstrated no evidence of current irAE such as HLH.

## Conclusion

To our knowledge, this is the first described case of pembrolizumab-induced HLH in HNSCC. The patient’s HLH was treated successfully with dexamethasone and etoposide. Due to disease progression while on observation pembrolizumab was restarted without complication to date. As the applications of immunotherapy continue to expand there will be more patients who suffer very rare side effects such as immunotherapy-induced HLH. Given the increasing numbers of case reports of immunotherapy-induced HLH as well as the lack of data regarding optimal treatment for HLH increased awareness of the potential development of HLH in patients receiving immunotherapy is clinically relevant and warrants further research.

## Data Availability

Not Applicable

## Abbreviations

HLH: Hemophagocytic Lymphohistiocytosis
HNSCC: Head and Neck Squamous Cell Carcinoma
irAEs: Immune-Related Adverse Events
PD-1: Programmed Cell Death-1
PD-L1: Programmed Cell Death-1 Ligand
